# CCND1-associated ceRNA network reveal the critical pathway of TPRG1-AS1-hsa-miR-363-3p-MYO1B as a prognostic marker for head and neck squamous cell carcinoma

**DOI:** 10.1038/s41598-023-38847-7

**Published:** 2023-07-22

**Authors:** Zehao Li, Xinguang Qiu, Qi He, Xinghao Fu, Feihong Ji, Xiufen Tian

**Affiliations:** 1 Department of Pharyngolaryngology Head and Neck Surgery, The First Affiliated Hospital of Zhengzhou University, Zhengzhou, Henan China; 2 Department of Thyroid Surgery, The First Affiliated Hospital of Zhengzhou University, Zhengzhou, Henan China

**Keywords:** Head and neck cancer, Computational biology and bioinformatics, Oncology, Risk factors

## Abstract

Head and neck squamous cell carcinoma (HNSC) is one of the leading causes of cancer death globally, yet there are few useful biomarkers for early identification and prognostic prediction. Previous studies have confirmed that CCND1 amplification is closely associated with head and neck oncogenesis, and the present study explored the ceRNA network associated with CCND1. Gene expression profiling of the Head and Neck Squamous Cell Carcinoma (HNSC) project of The Cancer Genome Atlas (TCGA) program identified the TPRG1-AS1-hsa-miR-363-3P-MYO1B gene regulatory axis associated with CCND1. Further analysis of the database showed that MYOB was regulated by methylation in head and neck tumors, and functional enrichment analysis showed that MYO1B was involved in "actin filament organization" and "cadherin binding ". Immune infiltration analysis suggested that MYO1B may influence tumorigenesis and prognosis by regulating the immune microenvironment of HNSC. MYO1B enhanced tumor spread through the EMT approach, according to epithelial mesenchymal transition (EMT) characterisation. We analyzed both herbal and GSCALite databases and found that CCND1 and MYO1B have the potential as predictive biomarkers for the treatment of HNSC patients. RT-qPCR validated bioinformatic predictions of gene expression in vitro cell lines. In conclusion, we found a CCND1-related ceRNA network and identified the novel TPRG1-AS1-hsa-miR-363-3p-MYO1B pathway as a possible HNSC diagnostic biomarker and therapeutic target.

## Introduction

Every year, over 830,000 people worldwide are diagnosed with head and neck Squamous cell carcinoma, including over 43,000 die as a result of this disease^1^. The most commonly malignancies of the head and neck are head and neck squamous cell carcinomas (HNSC), which arise from the mucosal epithelium of the oral cavity, throat, and larynx. Squamous cell carcinomas of the head and neck are frequently linked to tobacco-derived carcinogen exposure and heavy alcohol usage^2^, and tumors of the oropharynx are linked to infection with oncogenic strains of human papillomavirus (HPV), primarily HPV-16, but also HPV-18 and other strains to a lesser extent^3, 4^. Due to the lack of early HNSC screening indicators, two-thirds of patients are diagnosed with intermediate to advanced disease, and the survival rate of patients with locally advanced disease is significantly lower than that of patients with early disease, ranging from a 5-year survival rate of 49% in patients with laryngeal squamous cell carcinoma to 25% in patients with hypopharyngeal squamous cell carcinoma^5^. Currently, the main treatments for HNSC are surgery, radiotherapy, chemotherapy, immunotherapy, and targeted therapy. The major treatment options for early restricted HNSC include surgical resection and radiation, and if cancer advances and metastasizes substantially to lymph nodes and other systemic organ tissues, chemotherapy and immunotherapy are effective systemic treatments for HNSC. Commonly used antitumor medications include cisplatin and docetaxel, along with PD-1 checkpoint inhibitors^6, 7^. However, pharmacological therapy frequently fails due to the cancer cells' drug resistance. The current clinical treatment of progressive head and neck Squamous cell carcinoma is not promising, therefore, the pathophysiology of HNSC must be understood in order to develop novel diagnostic, prognostic, and therapeutic resistance biomarkers for HNSC research.

Long non-coding RNAs (LncRNAs) safeguard chromosome integrity and are engaged in transcription, translation, and epigenetic modification^8–10^, while the imbalance in LncRNAs have yet been credited to ailments which include the cancer^11^. MicroRNAs (MiRNAs) can target and regulate mRNAs to govern biological processes and modulate gene expression, and dysregulation of miRNA expression is strongly associated with cancer-related dysfunction^12^. In recent years, the network of competitive endogenous RNAs (ceRNAs) has revealed a fresh device for RNA interactivity^13^, and the ceRNAs mechanism has been demonstrated in a number of different types of malignancies, for instance gastric cancer, liver cancer, and lung cancer^14–16^.

Cancer cell proliferation is allied to an imbalance in cell cycle protein expression^17^, and cyclin D1 protein cell cycle protein D1 (CCND1) acts synergistically with CDK4 and CDK6 to play a key role in the transition from G1 to S phase of cells^18^. In head and neck Squamous cell carcinoma, CCND1 amplification is allied to the progression of heterogeneous proliferative lesions to carcinoma in situ and is associated with a unfavorable clinical outcome^19^. Meanwhile, abnormal expression of CCND1 is allied to the development and poor prognosis of hepatocellular carcinoma, breast cancer, lung cancer, and lymphoma^20–23^. It has been reported that CCND1 is involved in the mechanism associated with CDK4/6 cell cycle kinase inhibitor resistance in estrogen receptor-positive breast cancer^24^ while silencing CCND1 was found to increase the ovarian cancer cells' susceptibility to olaparib medicines^25^.

Therefore in research work, gene expression profiles and clinical information from HNSC patients were gathered and thoroughly analyzed from the Cancer Genome Atlas database in developing a ceRNA network coupled with CCND1. The TPRG1-AS1-has-miR-363-3p-MYO1B regulatory network was distinguished by analysis for expression, survival, and correlation. Our finding adds to the understanding of the molecular mechanism of HNSC and comes up with new targets for the treating of HNSC.

## Results

### High expression of CCND1 in HNSC and its relationship with patient prognosis

**Figure 1:**
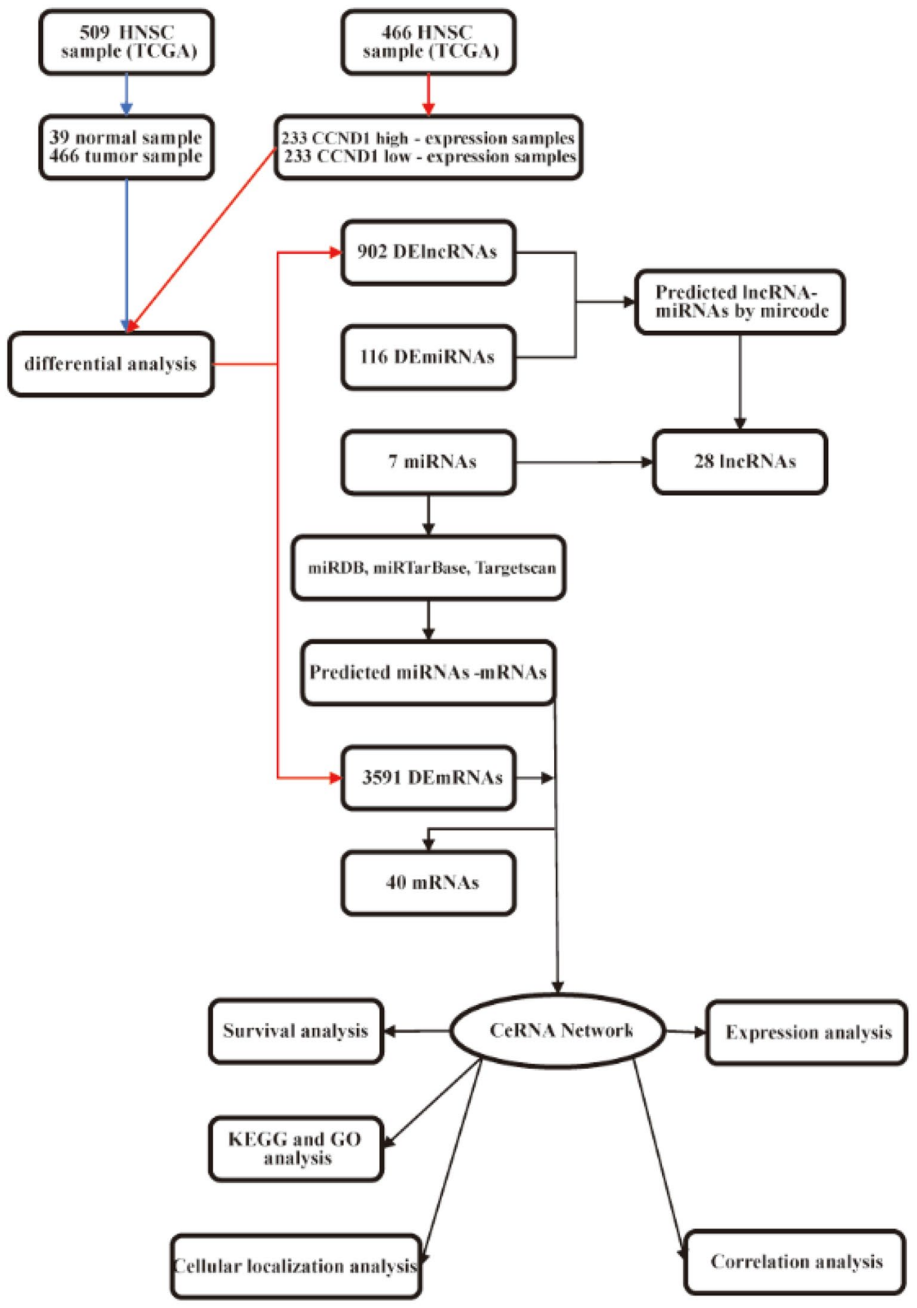
The flowchart of data collection and analysis.

**Figure 2:**
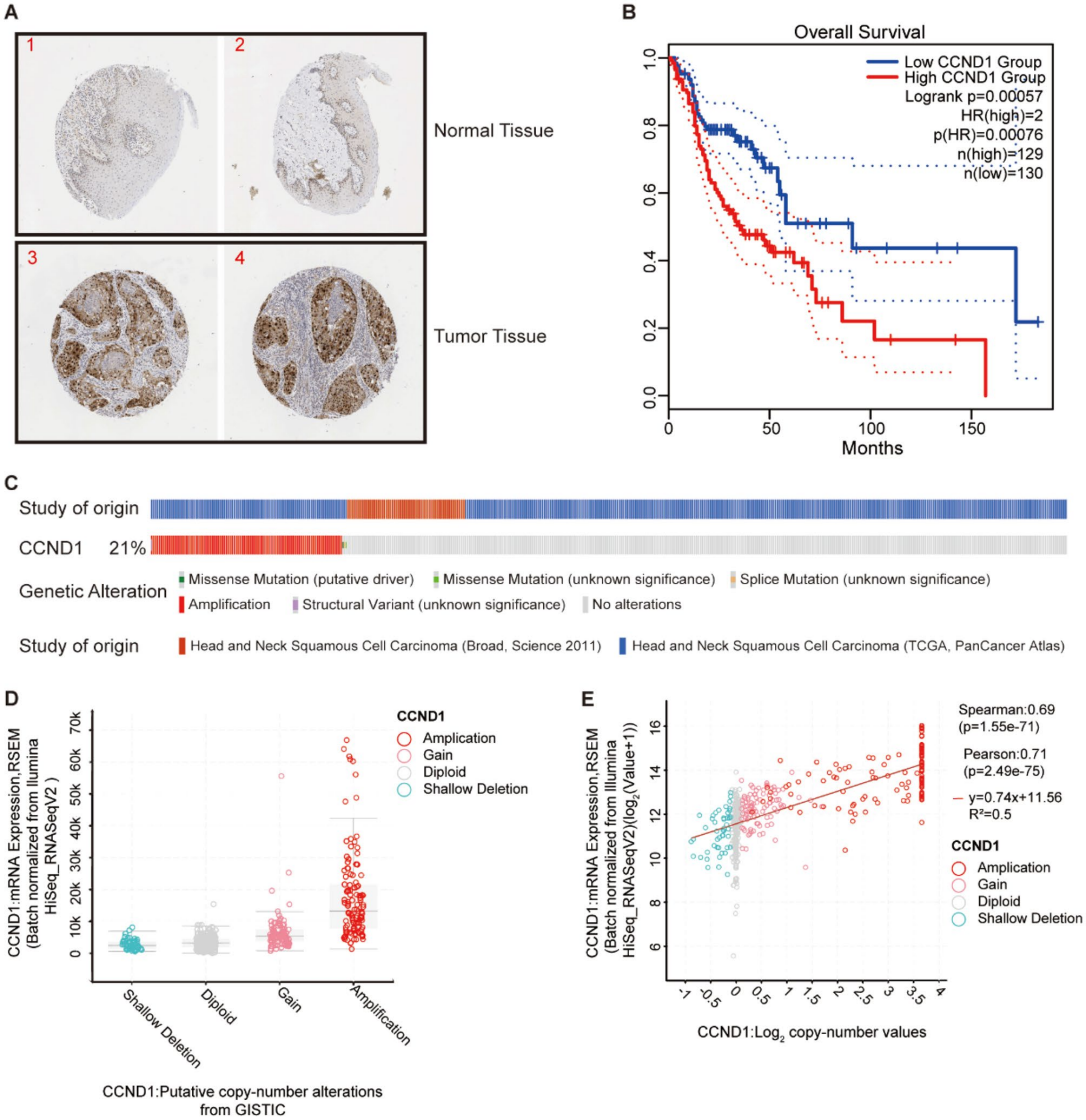
(**A**) CCND1 was expressed at higher levels in HNSC than in normal tissues by immunohistochemical analysis of the HPA database. (**B**) GEPIA2 database survival analysis data CCND1 overall survival rate. (**C**) The distribution of CCND1 genomic alterations in TCGA HNSC is shown on a cBioPortal OncoPrint plot. (**D**,**E**) The association between CCND1 copy number and mRNA expression are shown in the dot plot (**D**) and correlation plot (**E**) by cBioPortal.

The general workflow of this study is displayed in Fig. 1. The HPA database confirmed that CCND1 was significantly upregulated in terms of protein level in HNSC (Fig. 2A). The GEPIA2 database analysis revealed that elevated CCND1 expression reduced OS in HNSC, leading to a worse prognosis (Fig. 2B). To further explore the theorem of CCND1 high expression in HNSC, we went into the genome and copy number of CCND1 at the cBioPortal website, and the OncoPrint plot showed the amplification of CCND1 in the HNSC dataset (Fig. 2C). As depicted in Fig. 2D, the mRNA expression of CCND1 amplified HNSC samples was significantly higher than that of HNSC samples showing diploid CCND1 and CCND1 deletion. In addition, the copy number of CCND1 was discovered to be positively associated to mRNA expression in HNSC (Fig. 2E). In combination, the data suggest that CCND1 expression is elevated in HNSC, also the increase in CCND1 copy number may be the main mechanism leading to the upregulation of CCND1 expression in HNSC patients. In addition, combined with the results of clinical data analysis, CCND1 expression was significantly higher in smokers than non-smokers (Fig. S1A), CCND1 expression was greatly higher in drinkers than non-drinkers (Fig. S1B), and high CCND1 expression correlated with lymph node metastasis staging of HNSC (Fig. S1C). In conclusion, evidence suggest that CCND1 expression is upregulated in HNSC and is associated with tumor development.

### Construction of DERNAs and ceRNA networks of CCND1-related HNSC

**Figure 3:**
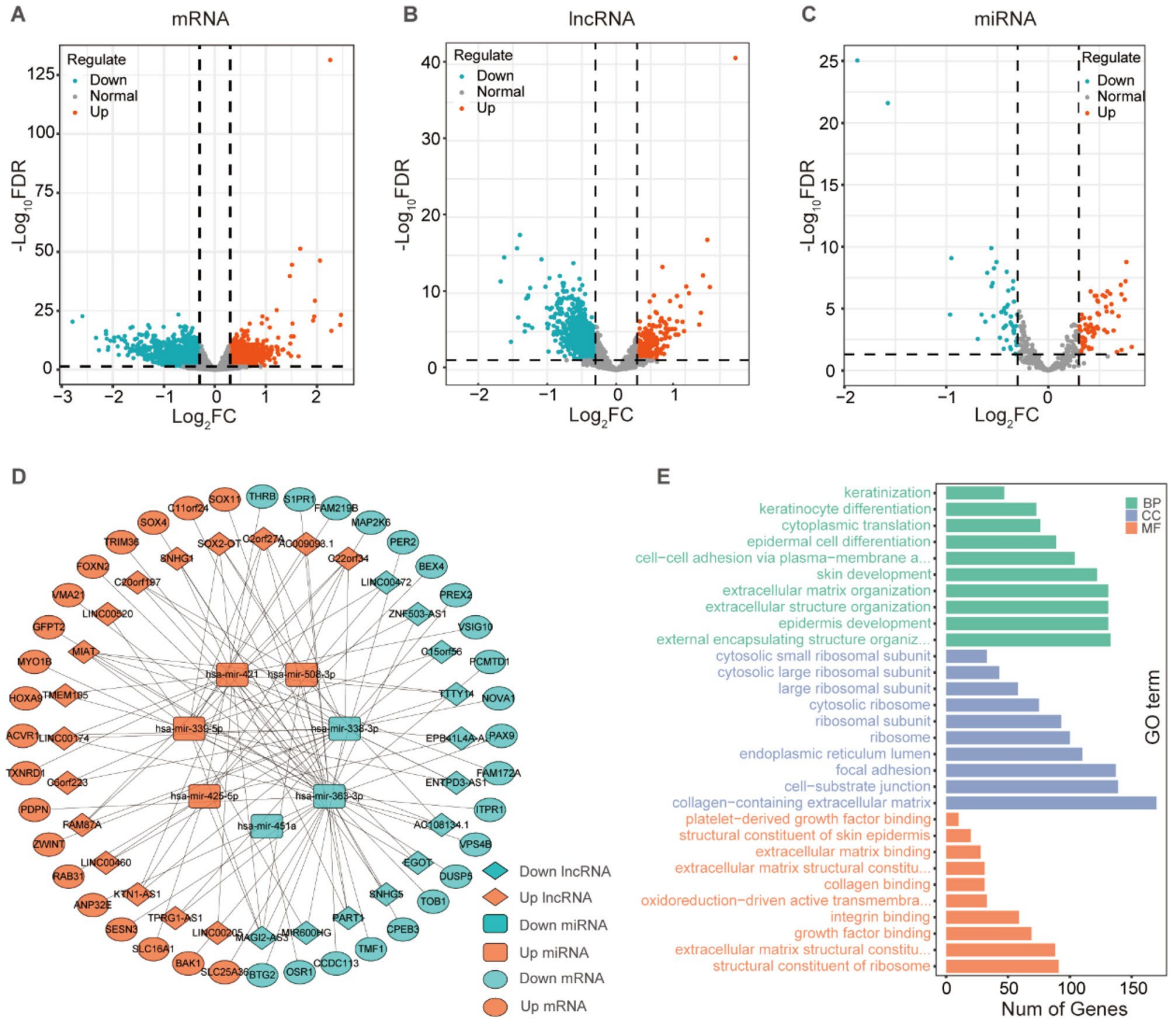
(**A**) The volcano map of 1438 upregulated and 2153 downregulated mRNAs. (**B**) The volcano map of 207 upregulated and 695 downregulated mRNAs. (**C**) The volcano map of 69 upregulated and 47 downregulated mRNAs. (**D**) A ceRNA network includes of 28 DElncRNAs, 7 DEmiRNAs and 40 DEmRNAs. (**E**) GO enrichment analysis of DEmRNAs.

The CCND1 expression was separated into group with poor expressiveness of CCND1 (n = 233) and group with high expressiveness of CCND1 (n = 233) according to the median of CCND1 expression, and the log2Fold change and FDR values of the distinct genes were measured using the R package "edgeR" to construct CCND1-related ceRNA network. By setting the lncRNA threshold|log2FC|> 0.3, miRNA threshold|log2FC|> 0.3 and mRNA threshold|log2FC|> 0.3, FDR values < 0.05 was declared statistically significant. A total of 902 lncRNAs, 116 miRNAs, and 3591 mRNAs were filtrated. Differential analysis Volcano plots were drawn using the R package "ggVolcano" (Fig. 3A–C).

To determine the DERNAs' regulatory interactions, the ceRNA network was established and displayed using Cytoscape in the light of miRcode, miRTarBase, TargetScan, and miRDB databases using 28 DElncRNAs, 7 DEmiRNAs, and 40 DEmRNAs (Fig. 3D). The R package "clusterProfiler" enrichment analysis reflected that the DEmRNAs were linked with "structural constituent of ribosome", "collagen-containing extracellular RNAs", and "collagen-containing extracellular RNAs". (Fig. 3E, Fig. S2A–C).

### Validation analysis of CCND1 related ceRNAs

**Figure 4:**
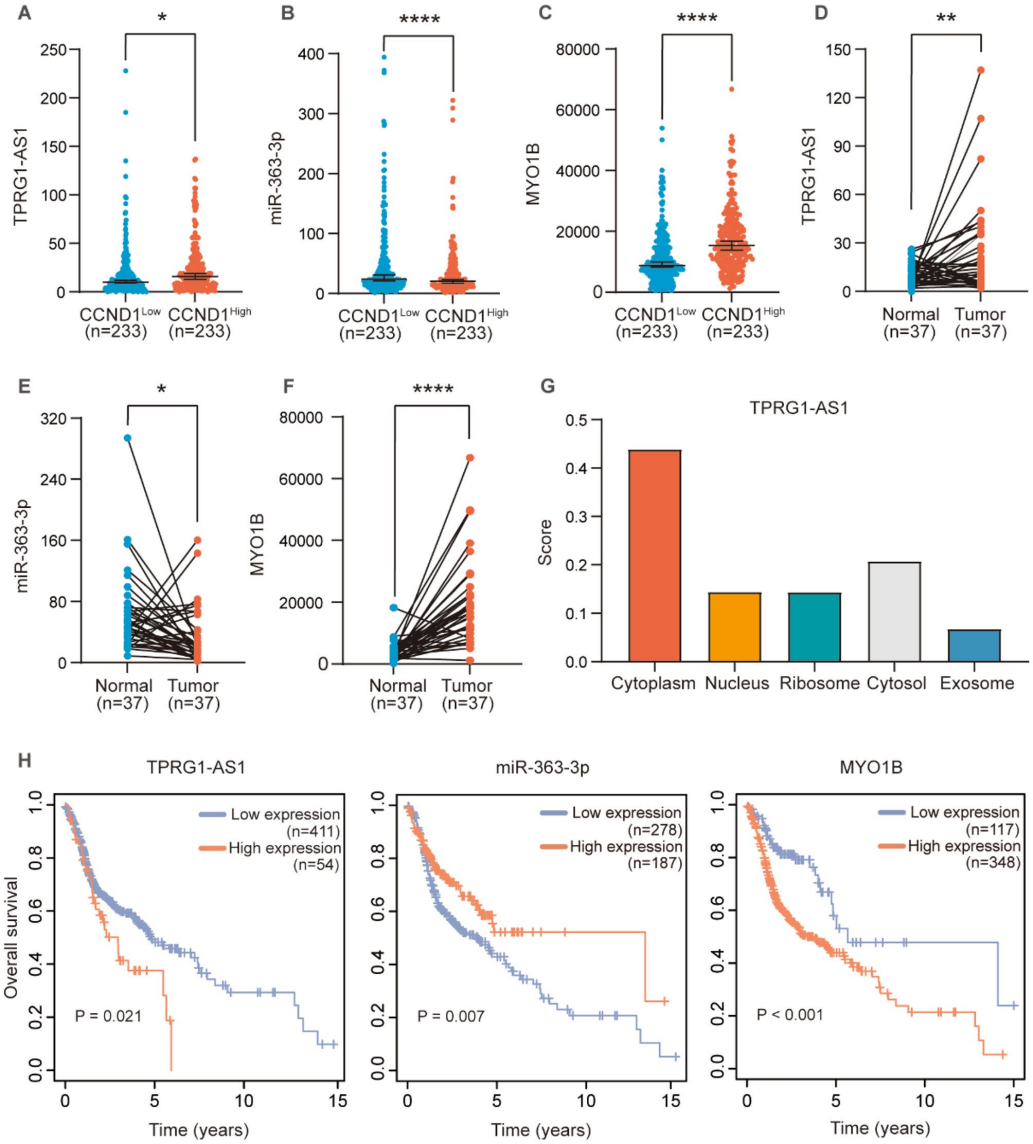
Expression level of DERNAs in CCND1low expression group and CCND1high expression group. (**A**) DElncRNA: TPRG1-AS1. (**B**) DEmiRNA: miR-363-3p (**C**) DEmRNA: MYO1B. Pairing of gene expression patterns between tumor tissue and normal tissue in patients with HNSC. (**D**) DElncRNA: TPRG1-AS1 (**E**) DEmiRNA: miR-363-3p (**F**) DEmRNA: MYO1B. (**G**) Subcellular localization of TPRG1-AS1. (**H**) The high- and low-expression values of DERNAs were compared by a Kaplan–Meier survival curve for TCGA HNSC patient cohort. *p < 0.05, **p < 0.01, ***p < 0.001, ****p < 0.0001.

**Figure 5:**
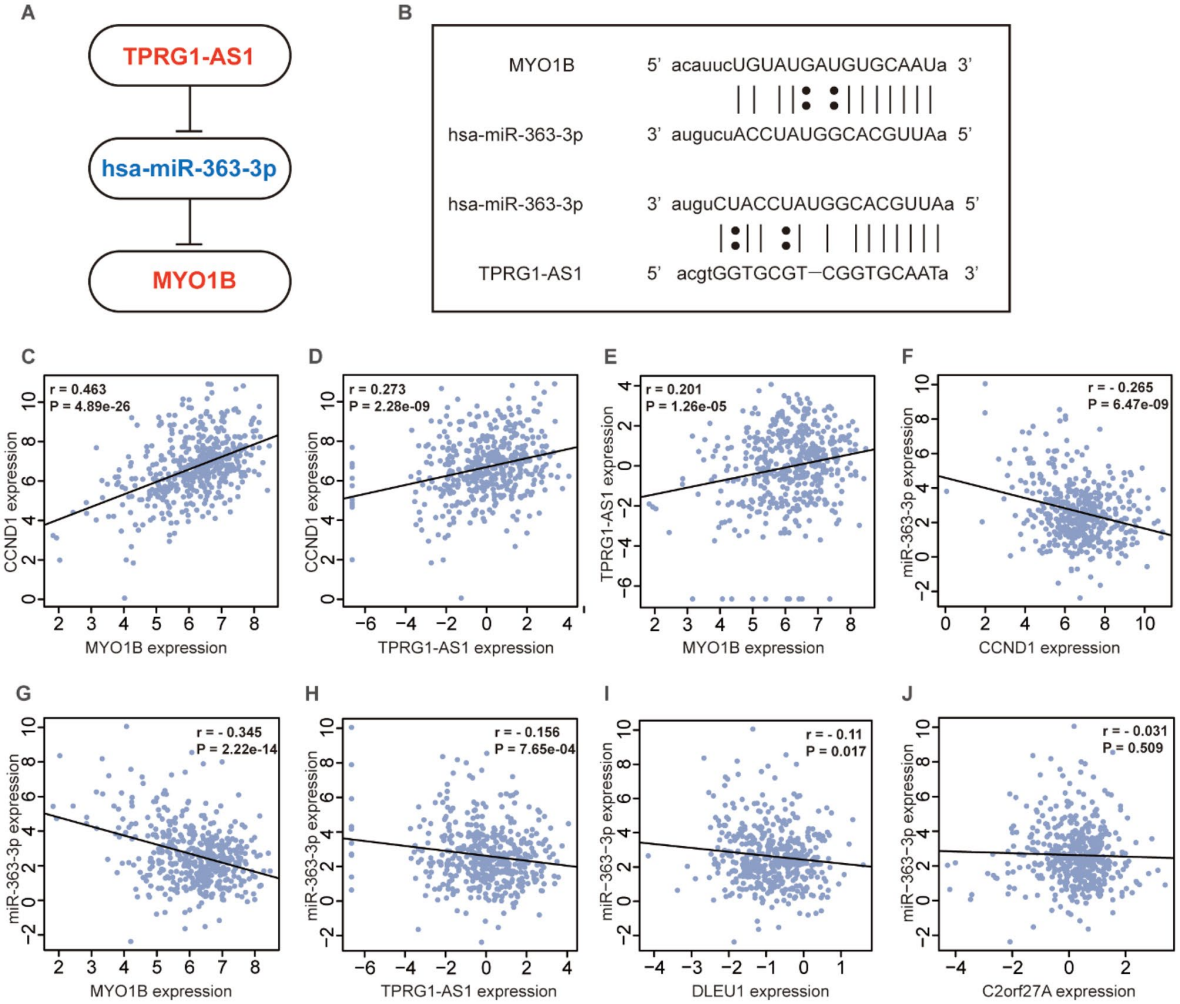
(**A**) Schematic model of ceRNA. Blue indicates downregulated; red indicates upregulated. (**B**) Base pairing between miR-363-3p and the target site in the TPRG1-AS1 and MYO1B predicted by miRanda and TargetScan, respectively. (**C**–**H**) Correlation analysis between these three predictive RNAs and CCND1 in HNSC, log2(RPM + 0.01) was used for miR-363-3p expression and log2(TPM + 0.01) was used for TPRG1-AS1, MYO1B, CCND1 expression.

To construct CCND1 expression-related ceRNAs, we scrutinized DERNAs expression levels in CCND1 low expression group and CCND1 high expression group, and FDR < 0.05 was defined as statistical significance (Fig. 4A–C, Fig. S3A,B). DERNAs expression levels in tumor tissues and matched paracancerous tissues from HNSC patients were also evaluated (Fig. 4D–F, Fig. S3C,D). And according to the one-way Kaplan–Meier regression analysis and LncRNA-miRNA-mRNA inter-matching, there were three DElncRNAs, one DEmiRNAs, and seven DEmRNAs to compound a ceRNA network closely connected to survival (Fig. 4H, Fig. S3E–G). After correlation analysis, the correlation between C2orf27A and hsa-miR-363-3P was not statistically significant (Fig. 5J), and the negative correlation between hsa-miR-363-3P and DLEU1 was statistically significant (Fig. 5I). The final correlation verified that hsa-miR-363-3p was inversely interacted with the expression of TPRG1-AS1, meanwhile TPRG1-AS1 was positively correlated with the mRNAs of MYO1B. Subcellular localization of DLEU1 and TPRG1-AS1 was analyzed at LncLocator online website, and the outcomes indicated that TPRG1-AS1 was localized in "Cytoplasm" (Fig. 4G) and DLEU1 was localized to "Cytosol" (Fig. S3H). The above results suggest that TPRG1-AS1 can increase the level of MYO1B through hsa-miR-363-3p sponge. Expression pairwise analysis of DERNAs and CCND1 showed that hsa-miR-363-3p was negatively correlated with CCND1. In contrast, TPRG1-AS1, MYOB, and CCND1 were positively correlated (Fig. 5C–H). ceRNAs base pairing results are shown in (Fig. 5A,B).

### Expression of ceRNAs in pan-cancerous tissues

To comprehend how prognostic models are expressed in pan-cancer, expression analysis of DERNAs was performed on TCGA and GTEx databases using the online database Xena Shiny (https://shiny.hiplot.com.cn/ucsc-xena-shiny/). As shown in Fig. S4, TPRG1-AS1 was upregulated in more than 1/3 of cancers, MYO1B in more than 2/3 of cancers, and hsa-miR-338-3p was downregulated in more than 1/3 of tumors.

### Methylation and protein expression levels of MYO1B

Genetic and environmental influences on human gene methylation levels^26^. To investigate the mode of MYO1B gene aberrant upregulation in HNSC, the UALCAN database was employed to detect the methylation levels. The outcomes demonstrated that the methylation level of MYO1B in HNSC was considerably lower than that in normal cells (Fig. S5A). DNA methylation is an influential DNA modification that governs gene silence^27^. Methylation sites (cg18731811, cg20700762, cg09973663, cg22768676, cg24376339, cg02119693, cg03134230, cg22885000, cg09760210) were positively correlated with MYO1B expression, and methylation sites (cg03796659, cg15096140, cg25475516, cg17167076, cg27659841, cg20391764, cg12404831, cg12738764, cg06844526) were negatively correlated with MYO1B expression. It was also found that most of the methylation sites negatively associated with MYO1B expression were located in the promoter region, and those positively associated with MYO1B expression were located within the gene body (e.g., Fig. S5C,D). The above results deduce that methylation may actively engage in the regulation of MYO1B expression, which then affects the development of HNSC.

Based on the UALCAN database, we found that MYO1B protein levels were upregulated in HNSC (Fig. S5B).

### Immune infiltration analysis

**Figure 6:**
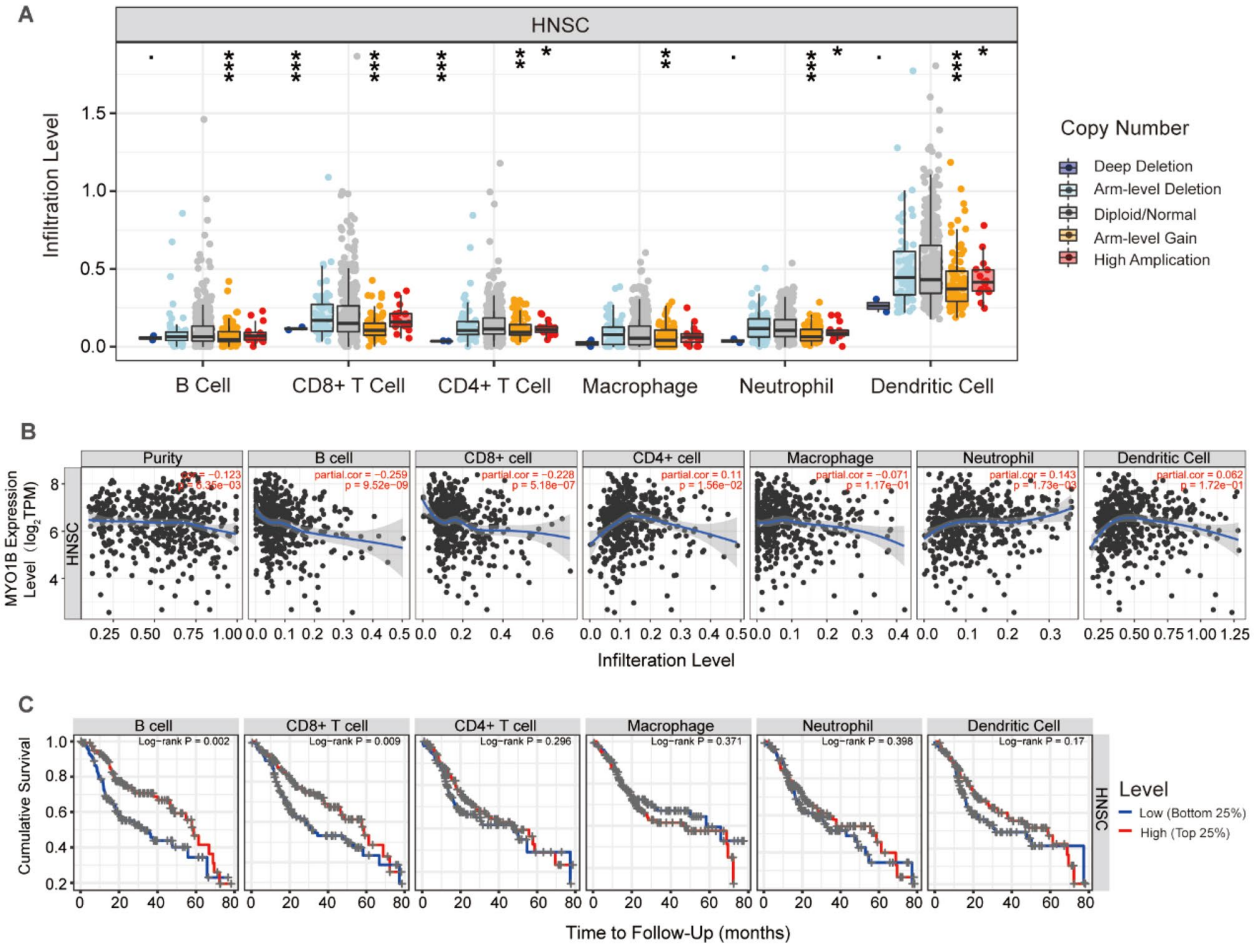
(**A**) Association between MYO1B gene copy number and immune cell infiltration levels in HNSC cohorts. (**B**) Correlation of CCND1 expression with immune infiltration level in HNSC. (**C**) Kaplan–Meier plots were used to analyze the immune infiltration and overall survival rate of HNSC. *p < 0.05, **p < 0.01, ***p < 0.001, ****p < 0.0001.

In order to investigate the immune infiltration level of MYO1B in tumors, the association with both gene copy number and immune cell infiltration level was first evaluated using the "SCNA" module (see Fig. 6A). The "Gene" module showed that MYO1B expression was observed to be negatively connected with B cells and CD8 + T cells, but favorably correlated with CD4 + T cell and neutrophil infiltration (Fig. 6B). The "Survival" module was combined to detect the prognostic correlation between the level of MYO1B gene-related B-cell and CD8 + T-cell infiltration in HNSC patients (Fig. 6C). Finally, the "Correlation" module was employed to investigate the link between MYO1B and 16 immune cell markers (Table S1), and the consequences inferred that MYO1B expression was adversely affected to some markers of CD8 + T cells, T cells, B cells, NK cells, and also with M2 Macrophage, Treg cell partial markers were positively correlated.

### Epithelial mesenchymal transition (EMT) analysis

EMT is a key procedure in tumor advance, tissue repair, and gains an aggressive phenotype and has a significant impact on tumor invasion, migration, and metastasis^28^. The loss of epithelial markers and the development of mesenchymal markers are considered signs that cells undergo EMT. We checked MYO1B levels with EMT markers across this study (Fig. S6A–C). MYO1B was shown to be inversely associated to the epithelial marker mucin-1 (MUC1) and positively tied to the mesenchymal indicators N-calcineurin (CDH2) and fibronectin (FN1). It is suggested that MYO1B may accelerate tumor growth by triggering EMT.

### MYO1B-associated gene enrichment analysis and interaction network

To further investigate the possible functions of MYO1B in HNSC, we analyzed the top 200 MYO1B-related genes in HNSC using GO and KEGG pathways (Fig. S6D–G). The enrichment associated with MYO1B was "regulation of the actin cytoskeleton". In addition, GO enrichment analysis related to MYO1B, including biological process (BP), cellular component (CC), and molecular function (MF), focused on "actin filament organization", "cadherin binding", "actin binding" and "actin filament binding".

**Figure 7:**
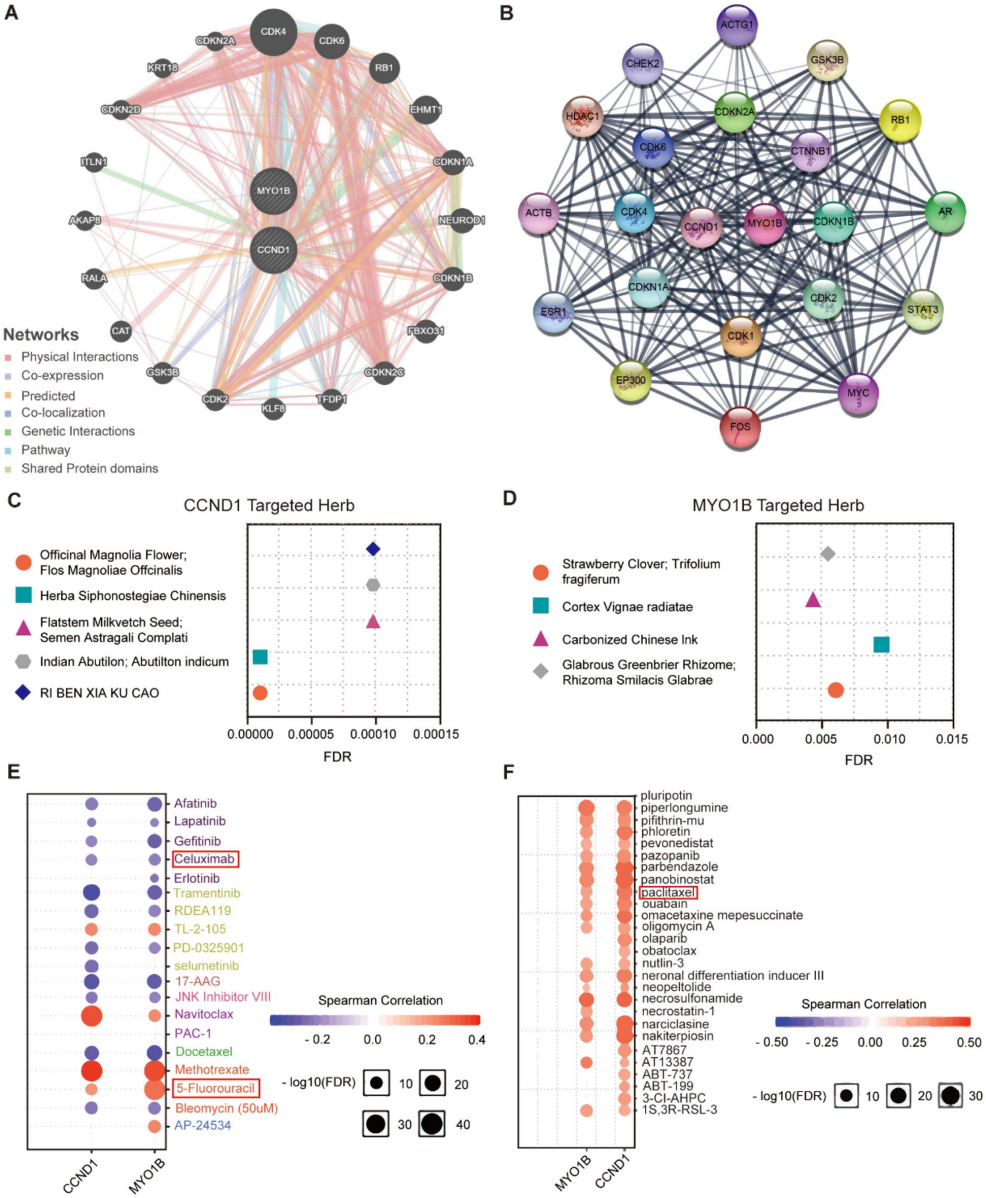
(**A**) Functional association networks of CCND1 and MYO1B. (**B**) Top 20 protein interaction networks of CCND1 and MYO1B; line thickness indicates the strength of data support. (**C**) The five most relevant herbs targeting CCND1. (**D**) Herbs targeting MYO1B. (**E**,**F**) Analysis of the correlation between CCND1, MYO1B and drug sensitivity based on (**E**) CTRP database and (**F**) GDSC database. Positive correlation implies that high expression of a gene is resistant to the drug, whereas negative correlation indicates sensitivity to this drug.

The functional relationship network of CCND1 and MYO1B was estimated by GeneMINIA, which observed 20 anticipate target genes (Fig. 7A). Meanwhile 20 potential candidate proteins were also displayed in the protein–protein interaction (PPI) network maps produced by STRING (Fig. 7B).

### Targeted herbal medicines and drug sensitivity analysis of CCND1 and MTO1B

Drug resistance remains a major limiting factor in the cure of cancer patients, and evidence suggests that despite tumors going into remission quickly, they develop drug resistance leading to disease recurrence^29^. HERB is a high-throughput database of experimental and reference guide herbal medicines for identifying 265 latent object herbs for CCND1, and 4 potential target herbs for MYO1B (Fig. 7C,D). In addition, the GSCALite database was explored based on the tumor treatment response portal (CTRP) and GDSC to assess the correlation of drug sensitivity with CCND1 and MYO1B. As shown in Fig. 7E,F, both CCND1 and MYO1B were resistant to "5-Fluorouracil" and "paclitaxel". In addition, high levels of expression of CCND1 and MYOB genes were sensitive to 11 drugs. Among them, "Cetuximab" targeted drugs have played an important role in the treatment of HNSC^30^.

### In vitro validation of CCND1-related regulatory genes

**Figure 8:**
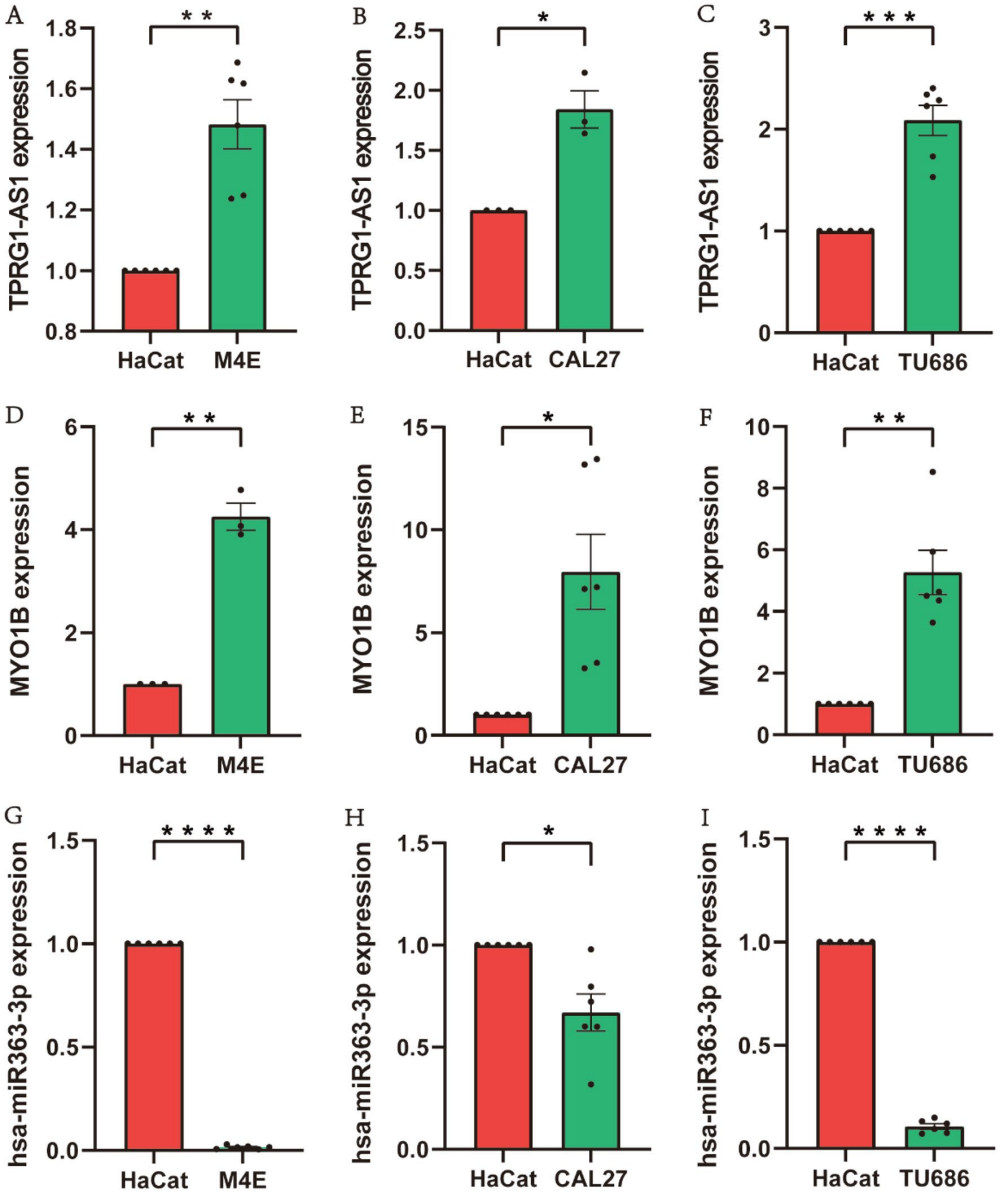
Verification of the three associated regulatory genes in HaCat cell and HNSC cells using quantitative real-time PCR, *p < 0.05, **p < 0.01, ***p < 0.001, ****p < 0.0001, compared with the HaCat cell. (**A**–**C**), TPRG1-AS1. (**D**–**F**), hsa-miR-363-3p. (**G**–**I**), MYO1B.

The bioinformatically predicted CCND1-related ceRNA regulatory genes were selected for RT-qPCR validation. The results revealed that the expression levels of lncRNA TPRG1-AS1 and mRNA MYO1B were significantly higher in HNSC cell lines than in HaCat cell (Fig. 8A–F), and the expression levels of miRNA miR-363-3p were significantly lower in HNSC cell lines than in HaCat cell (Fig. 8G–I). The consistent with the bioinformatic expression pattern, implying some reliability of the bioinformatic analysis results.

## Discussion

Advances in medical technology have led to significant advances in diagnostic and therapeutic techniques for HNSC, nevertheless relevant researches have declared that the overall survival rate of HNSC has not released^31^. For HNSC monitoring and therapy, it is essential to explore the molecular mechanisms of development. CeRNAs have a contribution to the activation of gastric, hepatocellular, and lung cancers^14–16^. To identify novel tumor biosignatures expressed in HNSC, we established CCND1-related ceRNAs. cyclin D1 protein cell cycle protein D1 (CCND1) synergizes with CDK4 and CDK6 to play a crucial part in the transition of cells from the G1 to the S phase^18^. In head and neck cancer, amplification of CCND1 is linked to the appearance of heterogeneous proliferative lesions to cancer in situ, as well as a poor clinical outcome^19^.

In this study, the samples of screened HNSC patients were classified into two groups based on CCND1 levels: low and high in the TCGA database, and ceRNA networks associated to CCND1 were predicted using computation, screening, and inter-matching. DEmRNAs were enriched in "structural constituent of ribosome ", "extracellular matrix structural constituent" and "growth factor binding". Therefore, utilizing survival and correlation analyses, we identified a prognostic pattern for HNSC, as in TPRG1-AS1 (up-regulated)- hsa-miR-363-3p (down-regulated)- MYO1B (up-regulated).

TPRG1-AS1 is upregulated in HNSC and promotes tumorigenesis by upregulating MYO1B through hsa-miR-363-3p sponging. It has been shown that TPRG1-AS1 serves as a potential driver of non-coding RNA to promote hepatocellular carcinoma progression, and its sponging of miR-4691-5p and miR-3659 as ceRNA leads to RBM24 expression, which inhibits hepatocellular carcinoma progression by activating the mechanism of tumor apoptosis^32^. According to the reports, MiR363 low expression is associated with proliferation and invasion of gastric cancer cells^33^ and miR-363-3p interacts with LncRNA OIP5-AS1 and promotes hepatocellular carcinoma progression by upregulating SOX4^34^. Meanwhile, aberrant expression of MYO1B boosts HNSCC cell migration and cervical lymph node metastasis^35^. Myosin 1b is associated with early treatment failure in oral tongue squamous cell carcinoma, a subgroup of head and neck cancer^36^, and myosin 1b can be used as a biomarker for oral tongue squamous cell carcinoma to help early detection of tumor cells complementing existing surgical and histopathological techniques used to identify definitive surgical margins^37^. MYO1B has also been found to be strongly expressed in cervical cancer and to be tied with HPV infection, lymphatic metastasis, and case grading, while knockdown of MYO1B in vitro in tumor cells significantly reduced MMP1/MMP9 activity and substantially repressed the proliferation, migration and invasive ability of cervical carcinoma tumor cells^38^.

In our study, TPRG1-AS1 and MYO1B expression in HNSC tissues was substantially greater than in normal tissues, and survival analysis revealed that the up-regulation of TPRG1-AS1 and MYO1B was attributed to unfavourable prognosis. In contrast to TPRG1-AS1 and MYO1B, miR-363-3p was down-regulated in HNSC, and also indicated a worse prognosis.

Hypermethylated standards of DNA in the promoter region are related to transcriptional repression and can inactivate tumor suppressor genes^39^, although many CpG islands (CGI) are located in gene promoters, they are also located within gene bodies^40^. The methylation of CPG in the gene body and gene expression are positively correlated, according to related investigates^41^, in contrast to the negative phase of methylation in the promoter that regulates gene expression^42^. One study found elevated methylation levels in the MYO1B gene body in colorectal cancer^43^. This is consistent with the finding that MYO1B up-regulation was inversely connected with methylation in the promoter region and positively linked with methylation in the majority of gene bodies in this study, suggesting that MYO1B may be regulated by methylation levels in HNSC.

Comprehending the interaction between the tumor and host immune system is essential for discovering forecasting biomarkers, lowering medication resistance, and constructing novel therapeutic approaches^44^. The immune components of the HNSC microenvironment include tumor-infiltrating lymphocytes (TIL; including T cells, B cells, and natural killer (NK) cells) and myeloid cells (including macrophages, neutrophils, dendritic cells, and myeloid suppressor cells (MDSCs))^19^. Studies have shown that most HNSC microenvironments are highly immunosuppressive^45, 46^ and that tumor immunity in the tumor microenvironment is mainly mediated by antitumor-active cells (effector T (Teff) cells) and NK cells, whereas immunosuppression and tumor cell growth is mediated by immunosuppressive-active cells (regulatory T (Treg) cells), the MDSCs and M2 macrophages mediated. Conversely, increased numbers of Treg cells, MDSCs, neutrophils, or M2 macrophages were linked to advanced HNSCC or a poor prognosis. In the tumor microenvironment, elevated levels of CD8 + T cells and NK cells were attributed to the greater survival^47^. The results of this study showed that MYO1B was negatively correlated with B-cell and CD8 + T-cell infiltration, and patients with high infiltration levels of B-cells and CD8 + T-cells had a better prognosis, (see Fig. 5B). Meanwhile, MYO1B was negatively correlated with NK cell marker levels and favorable connection with Treg cell and M2 macrophage marker levels (see Table S1). It is suggested that MYOB is associated with immune infiltration of HNSC.

Epithelial mesenchymal transition (EMT) plays a part in tumorigenesis and increases tumor metastatic properties through enhanced invasiveness^48^. To understand whether MYO1B is associated with the EMT process. We correlated gene expression with epithelial mesenchymal markers to explore the mechanisms associated with MYO1B in HNSC. MYO1B was shown to be inversely connected with epithelial markers and favorably correlated with mesenchymal markers in the study. It is proposed that MYO1B may facilitated the propagation and metastasis of HNSC through the EMT process. Meanwhile, it has been recently reported that silencing of MYO1B alleviated the EMT potential of HNSC^49^.

Cetuximab combined with platinum-based fluorouracil as first-line chemotherapy for patients with recurrent or metastatic squamous cell carcinoma of the head and neck improved overall patient survival compared to platinum plus fluorouracil chemotherapy alone^50^. This study suggested that up-regulation of CCND1 and MYO1B both showed resistance to "5-Fluorouracil" and "paclitaxel" and sensitivity to "Cetuximab" targeting drugs, indicating the potential of CCND1 and MYO1B as predictive biomarkers for HNSC patients treatment. (Fig. 7E,F). We also identified multiple target herbs associated with CCND1 and MYO1B, which need to be further investigated in the treatment of HNSC.

Although the ceRNA-based TPRG1-AS1-hsa-miR-363-3p-MYO1B axis seems to be a viable predictive biomarker for medical applications, the study's shortcomings must be acknowledged. Firstly, the interaction between ceRNA network needs to be further verified through experiments. Secondly, the role and mechanism of TPRG1-AS1-hsa-miR-363-3p-MYO1B axis in HNSC should be experimentally further investigated.

In summary, We established a crucial TPRG1-AS1-hsa-miR-363-3p-MYO1B axis that is linked to the generation of HNSC and may provide novel targets for HNSC therapy.

## Materials and methods

### Expression of CCND1 in HNSC

The GEPIA2 database (http://gepia2.cancer-pku.cn/) "survival analysis" module was used for CCND1 gene survival analysis. Human Protein Atlas (HPA, http://www.proteinatlas.org/) was employed to analyze the protein expression level of CCND1 in head and neck cancer. The amplification status of CCND1 and the amplification and mutation status of MYO1B were obtained through the Cancer Genomics cBioPortal website (http: //www.cbioportal.org/).

### Data preparation for HNSC

RNAseq and miRNAseq data for HNSC were acquired from the TCGA database (https://portal.gdc.cancer.gov/). The criteria for exclusion of samples were ⑴ radiotherapy prior to histological diagnosis of HNSC, ⑵existence of tumor other than HNSC, ⑶absence of the any clinical data, and ⑷ unavailability of samples to match with RNA and miRNA samples in the database. In this investigation, 466 HNSC tissue samples were collected. Patients' clinical data were also collected from the TCGA database, and no ethics committee permission was required. This work followed the TCGA publishing guidelines.

### Gene expression analysis

In light of the preceding description, the advancement of HNSC was associated with enhanced CCND1 expression, as a possible predictive model for HNSC, we designed a CCND1-related ceRNA network. Based on CCND1 levels, HNSC tumor samples were separated into two groups: low expression (n = 233) and high expression (n = 233). The R package "edgeR" utilized to estimate the log2FC and FDR results for each gene. Thresholds for lncRNA (|log2FC|> 0.3), miRNA (|log2FC|> 0.3), and mRNA (|log2FC|> 0.3) were set, and the levels of these RNAs were judged statistically different when the FDR values were < 0.05. The R package "ggplot2" performed volcano mapping of differentially expressed RNAs (DERNAs).

Mircode (http://www.mircode.org/) was employed to forecast the interregulation between DElncRNAs and DEmiRNAs, at that time TargetScan (http://www.targetscan.org/), miRDB (http://www.mircode.org/) and miRTarBase (https://mirtarbase.cuhk.edu.cn/) databases to anticipate DEmiRNAs target genes and detect intersection with DEmRNAs. Cytoscape version 3.8.0 shows the CCND1-related ceRNA network.

### Construction of ceRNA network

Patients' clinical information was retrieved from the TCGA database. Then, the R package "survival" was utilized to commit a univariate Kaplan–Meier analysis of the data to assess the association in both genes and OS. Survival-associated DElncRNAs, DEmiRNAs, and DEmRNAs were utilized to produce the ceRNA network.

The expression levels of DERNAs in paired HNSC patients and normal tissues along with in CCND1 low and CCND1 high expression groups were analyzed to screen the ceRNA network associated with CCND1. As well, pairwise analysis of lncRNA-miRNA, miRNA-mRNA, lncRNA-mRNA, DEmRNAs-CCND1, lncRNA-CCND1, and miRNA-CCND1 carried out to reveal the expression correlation between DERNAs-CCND1. The potential mechanism is proven by the LncRNAs' cellular localization; LncLocator analyzed the subcellular localization of lncRNAs (www.csbio.sjtu.edu.cn/bioinf/lncLocator). Base pairing between TPRG1-AS1-hsa-miR-363-3p-MYO1B was predicted using miRanda and TargetScan.

### Methylation and protein expression and mutation analysis

One of the earliest modification processes found was DNA methylation. The methylation levels of target genes were examined using the UALCAN database (http://ualcan.path.uab.edu/) to elucidate the mechanism of aberrant overexpression of MYO1B in HNSC. Beta values indicated that DNA methylation levels were in the range of 0 (unmethylated) to 1 (fully methylated). Additionally, we explored for methylation sites and related differentially methylated areas using the MEXPRESS (http://mexpress.be) and METHURV (https://biit.cs.ut.ee/Methsurv/) databases. Dataset CPTAC from the UALCAN database (http://ualcan.path.uab.edu/) was used to analyze the target gene protein expression levels. The z-values indicate the standard deviation of the median between HNSC samples, while the Log2 spectral count rate values for samples were normalized within and between samples.

The BioPortal dataset (https://www.cbioportal.org/) covers a variety of data types, including somatic mutations, DNA methylation, and pathway enrichment, to assist in the analysis of multidimensional cancer gene datasets. Bioinformatic approach to observe MYO1B amplification and mutation in tumor tissues.

### Levels of immune infiltration

Timer (https://cistrome.shinyapps.io/timer/) is an website tool focused on the quantification of cellular immune infiltration. The "SCNA" module provides a comparative exploration of tumor infiltration levels between tumors with different somatic cell copy number variations of a specific gene. The "Survival" module shows the clinical significance of immune cells in tumors. In addition, the "Genes" module visualizes the relationship between genes of interest and immune penetration levels in various malignancies. Finally, the "Correlation" module predicts the association between the MYO1B and 16 immune cell markers.

### Functional enrichment analysis and interaction network

To illustrate the biological processes and pathways that may be implicated in the HNSC prognostic model, the top 200 genes connected to CCND1 and MYO1B were gathered from GEPIA2 (gepia2.cancer-pku.cn). We used the R package "ClusterProfiler" to enrich these genes for GO and KEGG pathways and chosen the five most significant pathways, which were visualized by the R package "ggplot2".

GeneMINIA (http://genemania.org/) provides information on co-expression, genetic interplay, protein–protein physical interactions, shared protein structural domains, co-localization, and pathways. A distinct scoring algorithm is utilized to forecast functional relationship networks of target genes. STRING (https://string-db.org/) is a database that maps interaction networks based on protein co-promotion activities.

### Targeted drug analysis

The Herbal Medicines Database (http://herb.ac.cn/) is a natural herbal medicine platform for querying possible target herbs. GSCAlite (http://bioinfo.life.hust.edu.cn/web/GSCALite/) is a gene set tumor analysis platform. The interconnection of 481 drug probes in the CTRB gene with genes was analyzed using Spearman. Furthermore, the same exploration was also carried out on genes and over 100 pharmaceuticals in the Genomics of Drug Sensitivity in Cancer (GDSC) database.

### In vitro cellular RNA extraction and validation of quantitative real-time PCR

One human immortalized epidermal cell line (HaCat) and three HNSC cell lines (M4E, CAL27 and TU686) were identified by STR. M4E, TU686 cells were cultured with RPMI1640 containing 10% fetal bovine serum (Gibco, Detroit, MI, USA) and penicillin (100 units/ml, Grand Island, NY, USA) and streptomycin (100 μg/ml, Grand Island, NY, USA). USA) and streptomycin (100 μg/ml, Grand Island, NY, USA) in RPMI1640 culture medium, HaCat, CAL27 cells were treated with 10% fetal bovine serum (Gibco, Detroit, MI, USA) and penicillin (100 units/ml, Grand Island, NY, USA) and streptomycin (100 μg/ml, Grand Island, NY, USA) in DMEM culture medium. Cells were incubated in a humidified environment in a constant temperature and humidity incubator at 37◦ C containing 5% CO2 and 95% air, and trypsin digested. Total RNA was extracted using FastPure Cell/Tissue Total RNA Extraction Kit (Vazyme, Nanjing, Jiangsu, China). Reverse transcription was performed according to the instructions of HiScript® II Reverse Transcriptase Reagent (Vazyme, Nanjing, Jiangsu, China). Quantitative polymerase chain reaction (Q-PCR) was performed with ChamQ Universal SYBR qPCR Master Mix reagent (Vazyme, Nanjing, Jiangsu, China) according to the instructions. GAPDH and U6 were used as internal reference genes. Relative expression was calculated using the ΔΔ-Ct method. The primer sequences are shown in Table S2.

### Statistical analysis

Statistical analyses were performed using R software (https://www.r-project.org/, version 4.2.0). Cytoscape version 3.8.0 visualized the data using univariate Kaplan–Meier analysis to reveal the connection between gene expression and patient OS. p < 0.05 was judged statistically significant. The edgeR package was applied for the variance analysis and FDR < 0.05 was declared statistically significant.

## Supplementary Information

Supplementary Information.

## Data Availability

All data generated or analyzed during this study are included in this article. I uploaded the dataset and related code at the following URL: https://www.jianguoyun.com/p/DUtHc6gQg7uLCxisiOUEIAA Xena Shiny (https://shiny.hiplot.com.cn/ucsc-xena-shiny/), GEPIA2 (http://gepia2.cancer-pku.cn/), BioPortal (https://www.cbioportal.org/), TCGA (https://portal.gdc.cancer.gov/), Mircode (http://www.mircode.org/), TargetScan (http://www.targetscan.org/), miRDB (https://mirdb.org/), miRTarBase (https://mirtarbase.cuhk.edu.cn/), LncLocator (www.csbio.sjtu.edu.cn/bioinf/lncLocator), UALCAN (http://ualcan.path.uab.edu/), MEXPRESS (http://mexpress.be), Timer (https://cistrome.shinyapps.io/timer/), GeneMINIA (http://genemania.org/), STRING (https://string-db.org/), Herbal Medicines (http://herb.ac.cn/), GSCAlite (http://bioinfo.life.hust.edu.cn/web/GSCALite/).

## Abbreviations

HNSC: Head and neck squamous cell carcinoma
EMT: Epithelial mesenchymal transition
ceRNA: Competitive endogenous RNA
TCGA: The cancer genome atlas
DElncRNAs, DEmiRNAs, DEmRNAs: Differentially expressed lncRNAs/miRNAs/mRNAs
GO: Gene ontology
KEGG: Kyoto encyclopedia of genes and genomes
