# Supplementary material for: CCND1-associated ceRNA network reveal the critical pathway of TPRG1-AS1-hsa-miR-363-3p-MYO1B as a prognostic marker for head and neck squamous cell carcinoma
Source: Sci Rep. 2023 Jul 22;13:11831. doi: 10.1038/s41598-023-38847-7 (PMC10363142; doi:10.1038/s41598-023-38847-7)
Supplement: Supplementary file 1 — Supplementary Information. [file 41598_2023_38847_MOESM1_ESM.pdf]

CCND1-associated ceRNA network reveal the critical pathway of TPRG1-AS1-hsa-miR-363-3p-MYO1B as a prognostic marker for head and neck squamous cell carcinoma

Zehao Li[1], Xinguang Qiu[2*], Qi He[2], Xinghao Fu[2], Feihong Ji[2], Xiufen Tian[1]

[1]Department of Pharyngolaryngology Head and Neck Surgery, The First Affiliated Hospital of Zhengzhou University, Zhengzhou City, Henan Province, China

[2]Department of Thyroid Surgery, The First Affiliated Hospital of Zhengzhou University, Zhengzhou, Henan Province, China

* Correspondence:

Corresponding Author

qxg0914@126.com

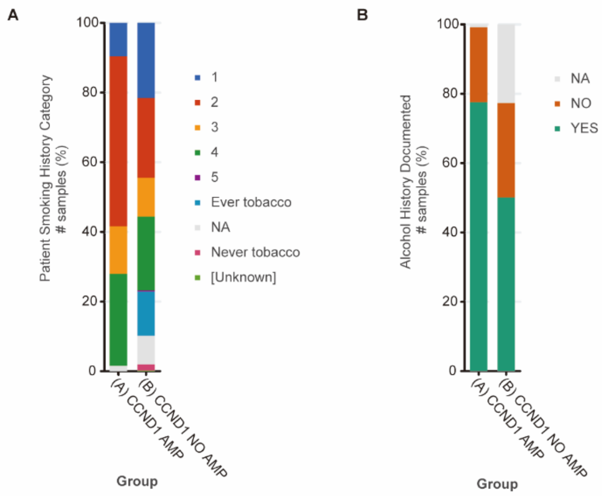

Figure S1: A. Smoking status of HNSC patients in the CCND1 amplification group versus HNSC patients in the no-amplification group in the cBioPortal database. B. Alcohol consumption in HNSC patients in the CCND1 amplification group versus HNSC patients in the no-amplification group in the cBioPortal database. C. Cervical lymph node metastasis in HNSC patients in the CCND1 amplification group versus HNSC patients in the no-amplification group in the cBioPortal database.

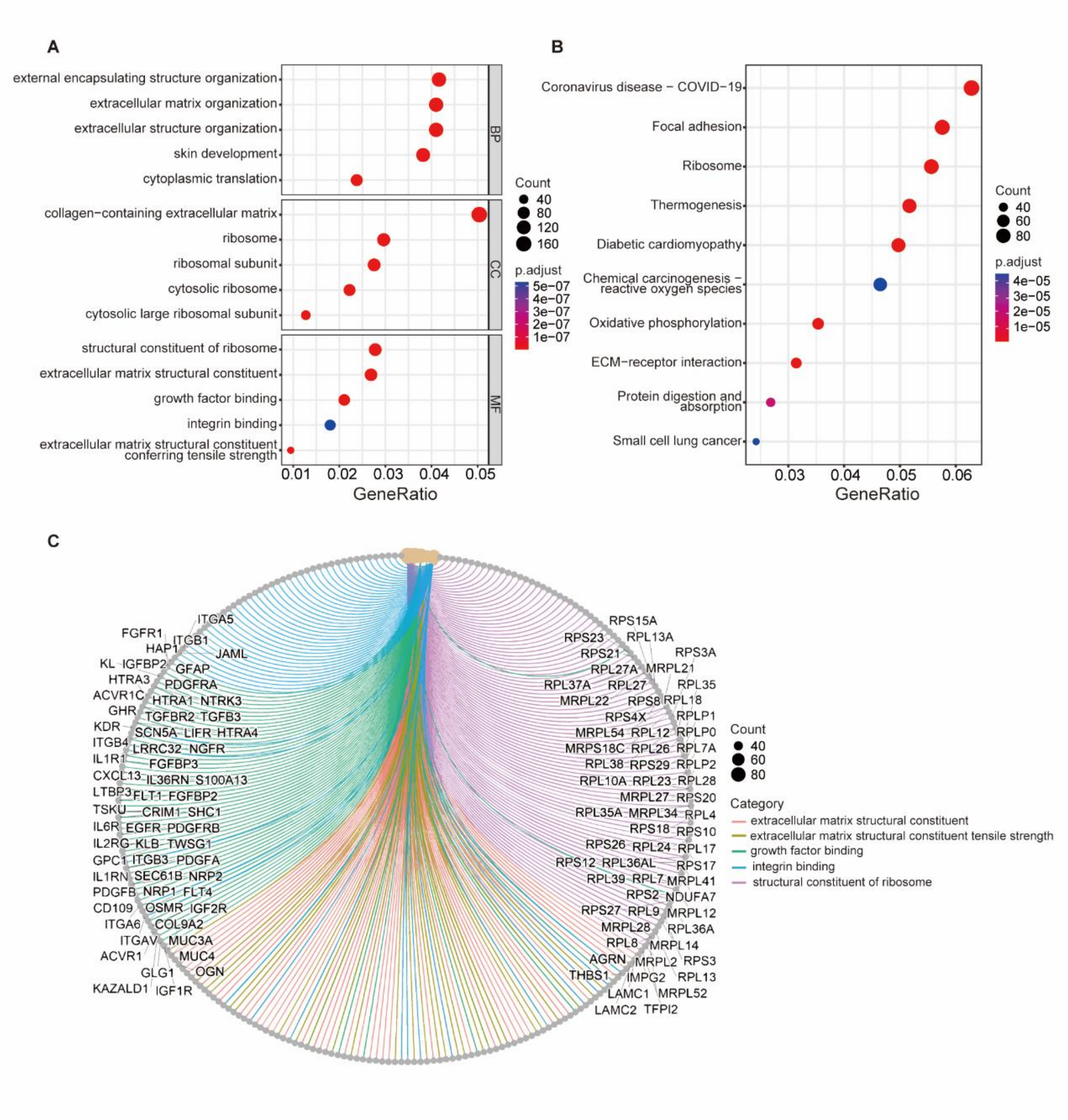

Figure S2: A. GO enrichment analysis of DEmRNAs. B. KEGG enrichment analysis of DEmRNAs. C. GO enrichment analysis of DEmRNAs for 5 related functions' genes

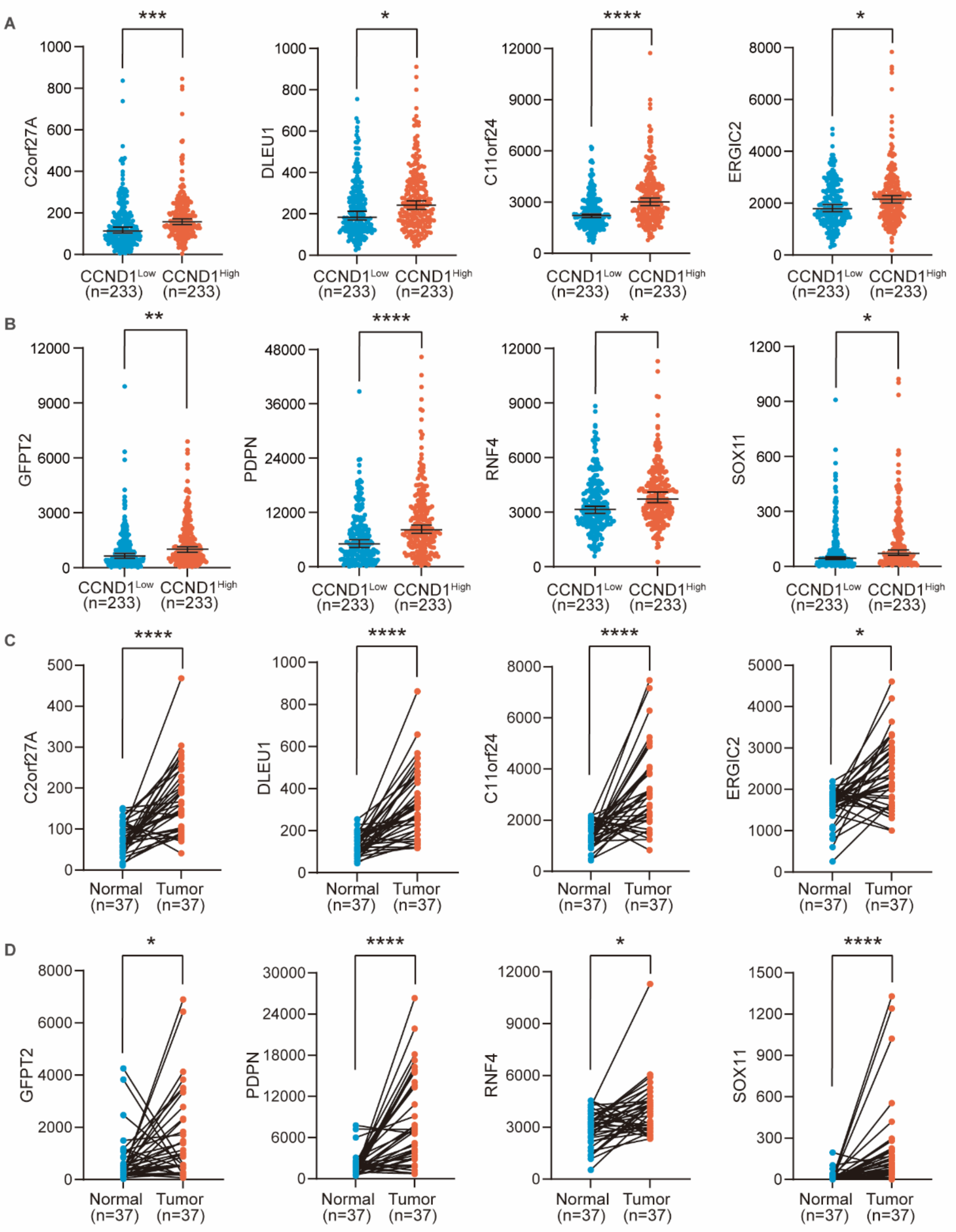
A
B
C
D
C2orf27A
DLEU1
C11orf24
ERGIC2
GFPT2
PDPN
RNF4
SOX11
CCND1Low (n=233)
CCND1High (n=233)
Normal (n=37)
Tumor (n=37)
***
*
****
**

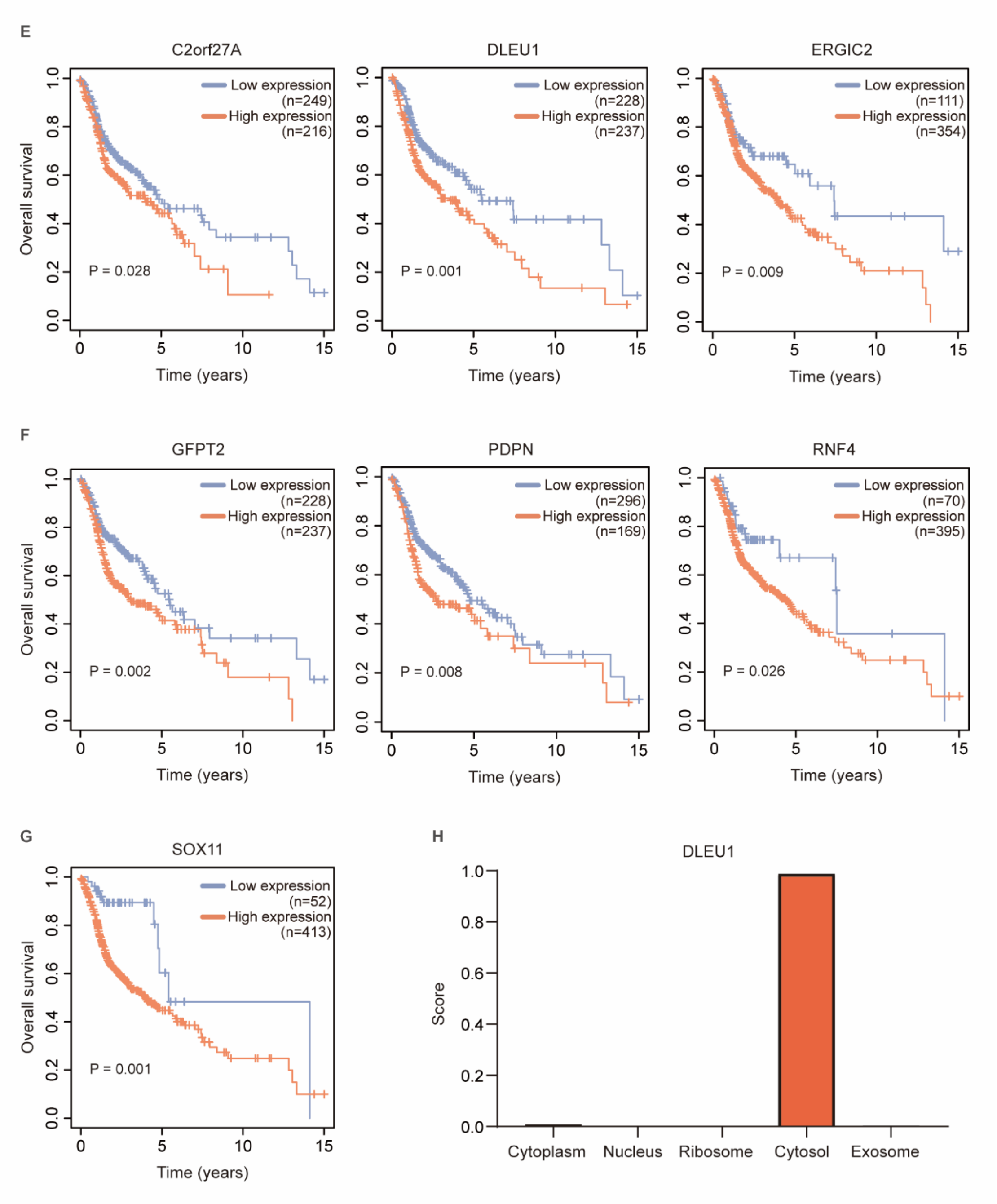

Figure S3: Expression level of DERNAs in CCND1low expression group and CCND1high expression group. (A) DElncRNA: C2orf27A, DLEU1. DEmRNA: C11orf24, ERGIC2 (B) DEmRNA: GFPT2, PDPN, RNF4, SOX11. Pairing of gene expression patterns between tumor tissue and normal tissue in patients with HNSC. (C) DElncRNA: C2orf27A, DLEU1. DEmRNA: C11orf24, ERGIC2 (D) DEmRNA: GFPT2, PDPN, RNF4, SOX11. *p < 0.05, **p < 0.01, ***p < 0.001, ****p < 0.0001. (E) Overall survival: C2orf27A, DLEU1, ERGIC2. (F) Overall survival: GFPT2, PDPN, RNF4. (G) Overall survival: SOX11. (H) Subcellular localization of DLEU1.

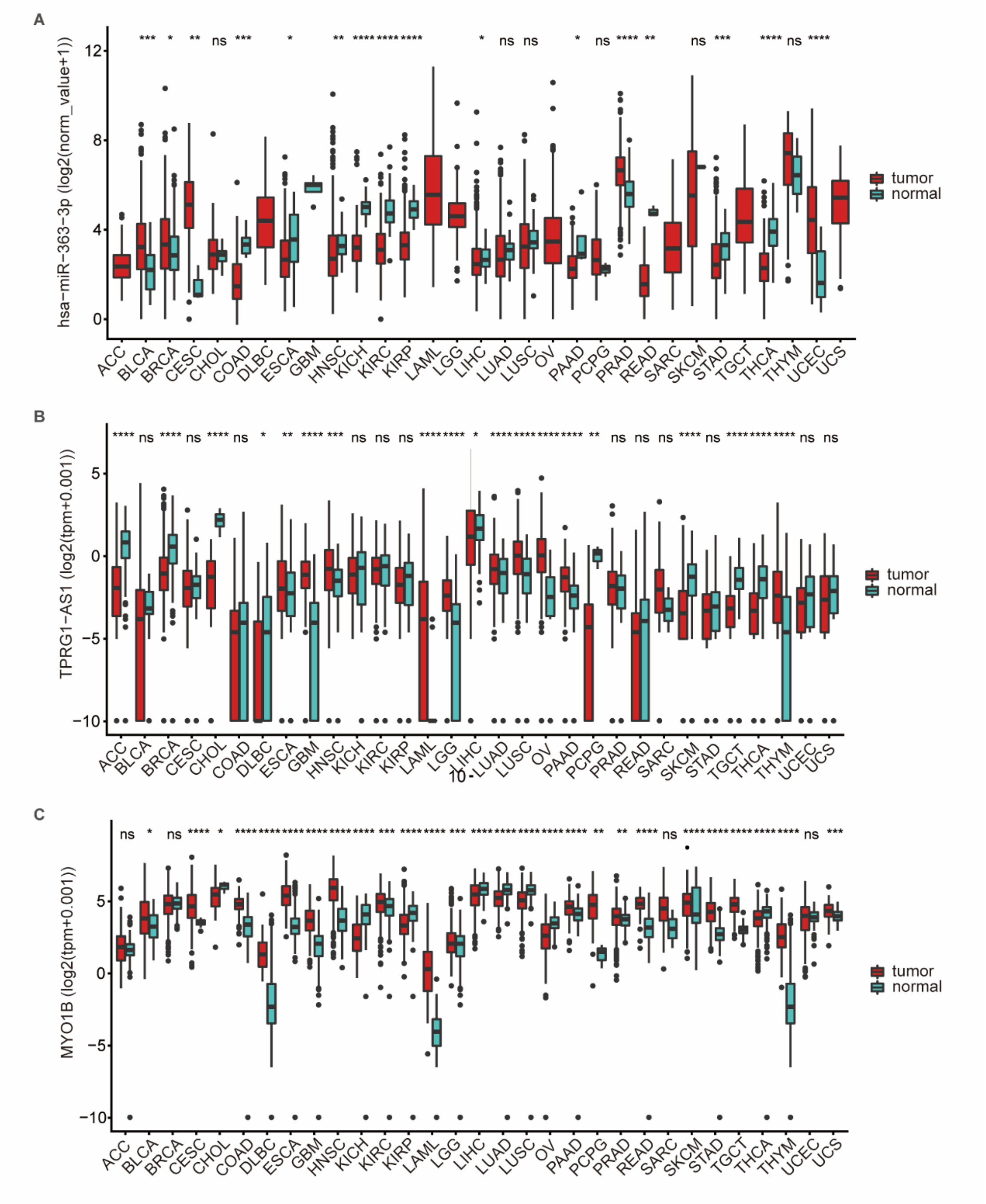

Figure S4: A. Expression analysis of has-miR-363-3p in normal tissues and pan−cancer samples. B. Expression analysis of TPRG1-AS1 in normal tissues and pan-cancer samples. C. Expression analysis of MYO1B in normal tissues and pan−cancer samples. *p < 0.05, **p < 0.01, ***p < 0.001, ****p < 0.0001

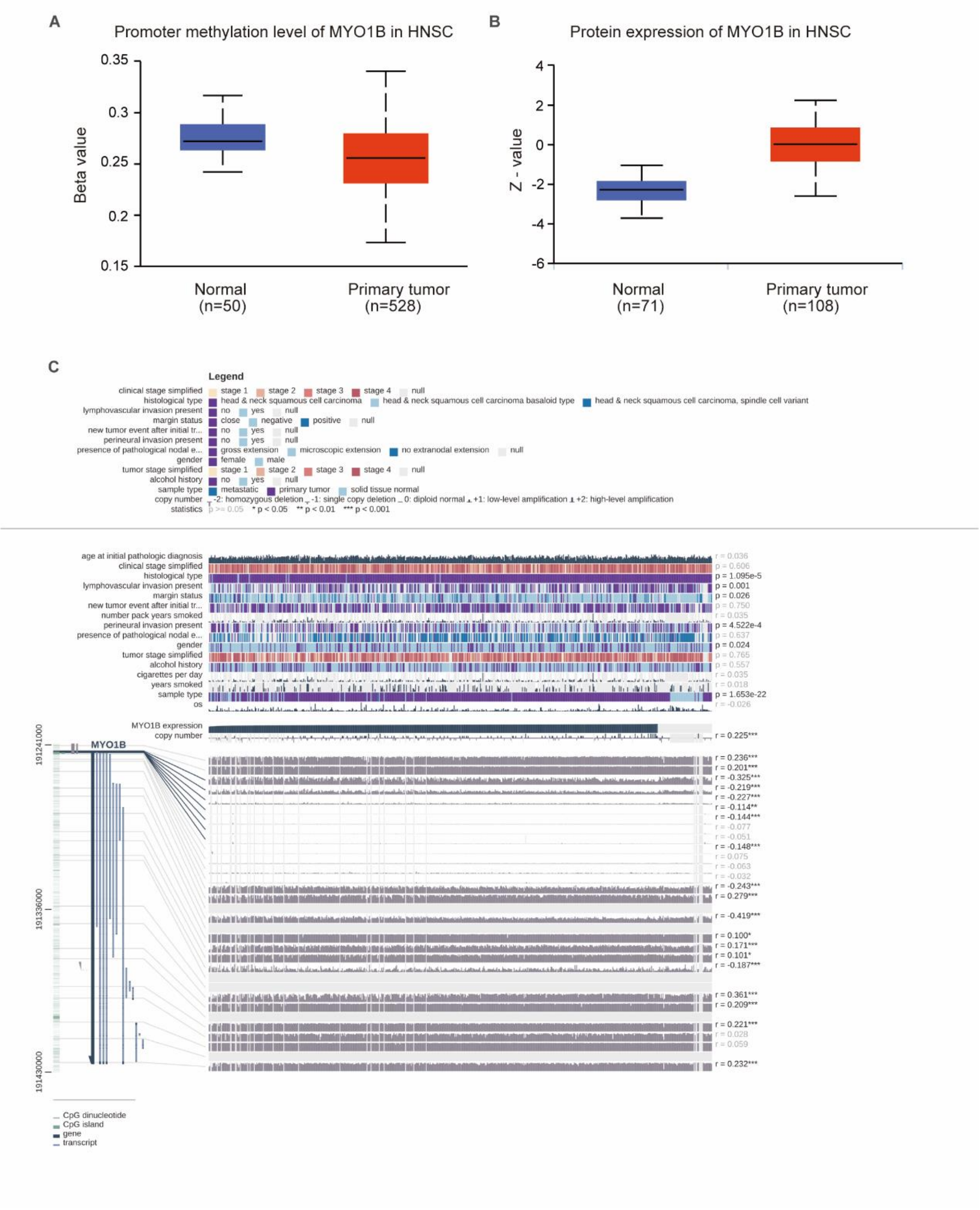
A
Promoter methylation level of MYO1B in HNSC
Beta value
0.35
0.3
0.25
0.2
0.15
Normal (n=50)
Primary tumor (n=528)
B
Protein expression of MYO1B in HNSC
Z - value
4
2
0
-2
-4
-6
Normal (n=71)
Primary tumor (n=108)
C
Legend
clinical stage simplified: stage 1, stage 2, stage 3, stage 4, null
histological type: head & neck squamous cell carcinoma, head & neck squamous cell carcinoma basaloid type, head & neck squamous cell carcinoma, spindle cell variant
lymphovascular invasion present: no, yes, null
margin status: close, negative, positive, null
new tumor event after initial tr...: no, yes, null
perineural invasion present: no, yes, null
presence of pathological nodal e...: gross extension, microscopic extension, no extranodal extension, null
gender: female, male
tumor stage simplified: stage 1, stage 2, stage 3, stage 4, null
alcohol history: no, yes, null
sample type: metastatic, primary tumor, solid tissue normal
copy number: -2: homozygous deletion, -1: single copy deletion, 0: diploid normal, +1: low-level amplification, +2: high-level amplification
statistics: p >= 0.05, * p < 0.05, ** p < 0.01, *** p < 0.001
age at initial pathologic diagnosis r = 0.036
clinical stage simplified p = 0.606
histological type p = 1.095e-5
lymphovascular invasion present p = 0.001
margin status p = 0.026
new tumor event after initial tr... p = 0.750
number pack years smoked r = 0.035
perineural invasion present p = 4.522e-4
presence of pathological nodal e... p = 0.637
gender p = 0.024
tumor stage simplified p = 0.765
alcohol history p = 0.557
cigarettes per day r = 0.035
years smoked r = 0.018
sample type p = 1.653e-22
os r = -0.026
MYO1B expression
copy number r = 0.225***
MYO1B
191241000
191336000
191430000
r = 0.236***
r = 0.201***
r = -0.325***
r = -0.219***
r = -0.227***
r = -0.114**
r = -0.144***
r = -0.077
r = -0.051
r = -0.148***
r = 0.075
r = -0.063
r = -0.032
r = -0.243***
r = 0.279***
r = -0.419***
r = 0.100*
r = 0.171***
r = 0.101*
r = -0.187***
r = 0.361***
r = 0.209***
r = 0.221***
r = 0.028
r = 0.059
r = 0.232***
CpG dinucleotide
CpG island
gene
transcript

D

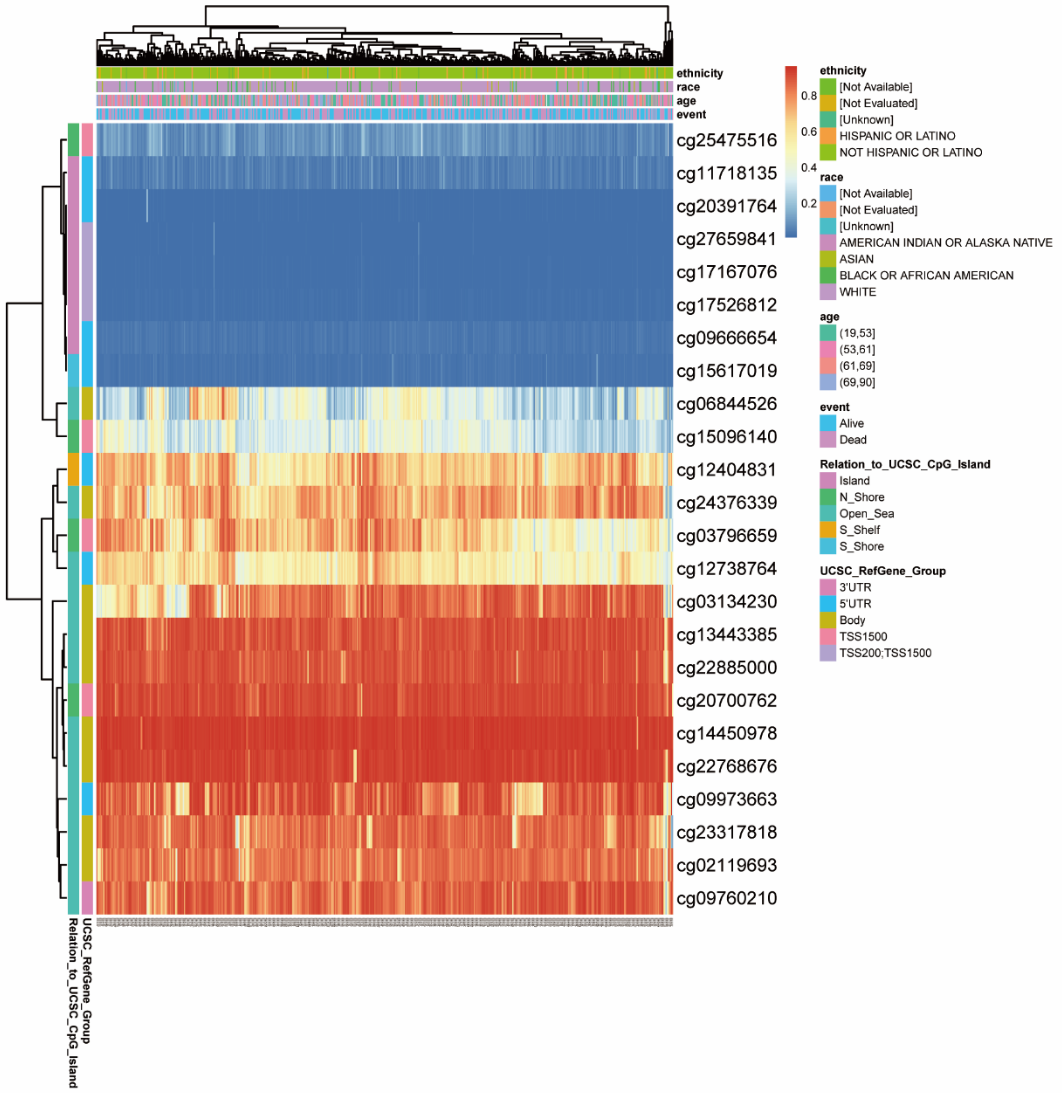

Figure S5: A. Methylation levels of MYO1B in HNSC. B. Protein expression of MYO1B in HNSC. C. Relationship between methylation sites and expression of MYO1B. D. Distribution of different methylation regions related to MYO1B

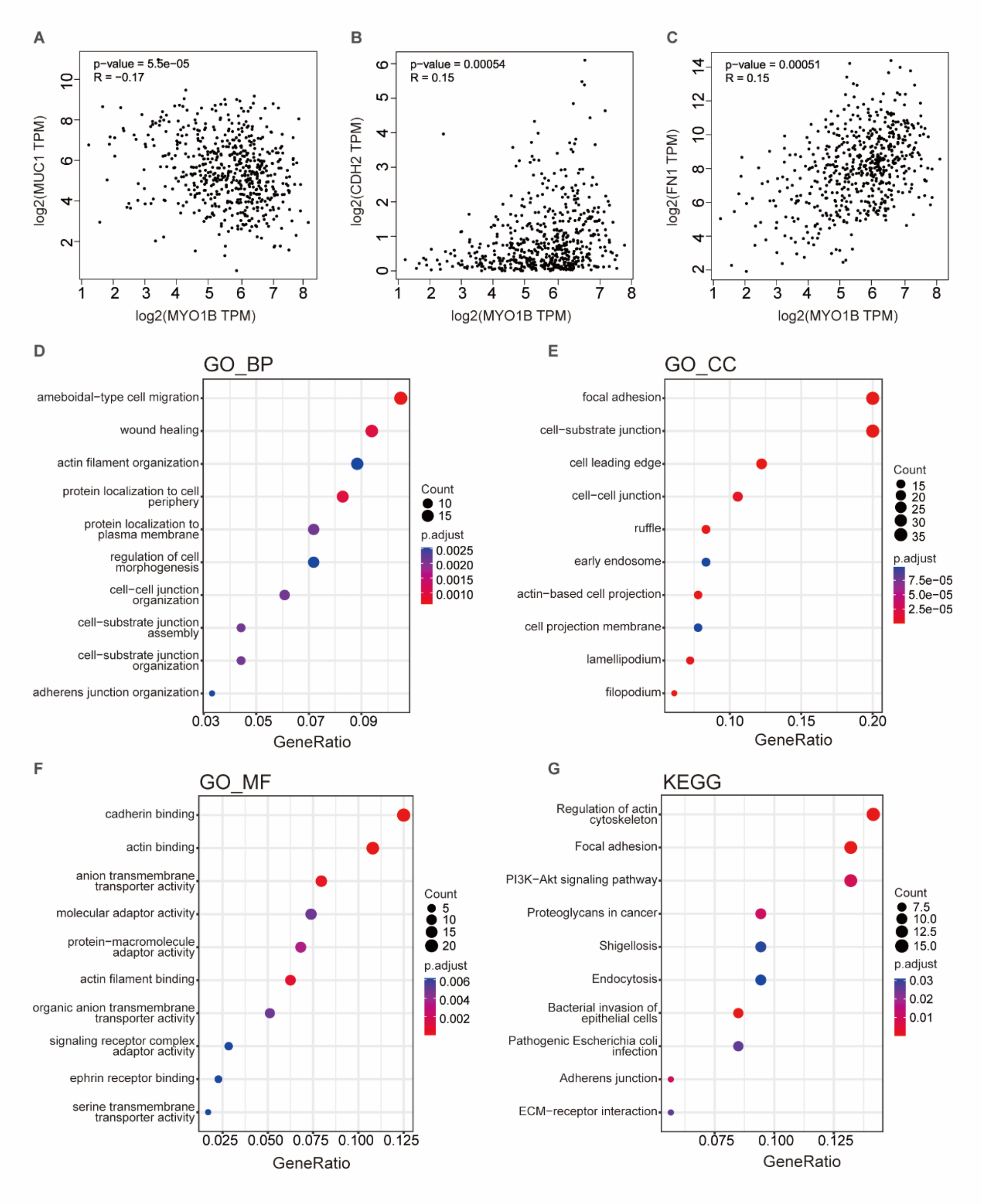

Figure S6: The expressions of MYO1B were associated with EMT makers (A) MUC1, (B) CDH2, (C) FN1. Enrichment analysis of MYO1B (D-G)

Description	Gene markers	HNSC-MYO1B	
		correlation	Pvalue
CD8+T cell	CD8A	-0.12319	0.004822
	CD8B	-0.20854	1.54E-06
T cell	CD3D	-0.20293	2.95E-06
	CD3E	-0.13358	0.002225
	CD2	-0.15254	0.00047
B cell	CD19	-0.27796	1.03E-10
	CD79A	-0.27203	2.62E-10
Monocyte	CD86	0.152252	0.000482
	CD115(CSF1R)	0.067013	0.126233
TAM	CCL2	0.041468	0.344364
	CD68	0.164391	0.000162
	IL10	0.17749	4.55E-05
M1 Macrophage	INOS(NOS2)	-0.25476	3.54E-09
	IRF5	-0.19534	6.93E-06
	COX2(PTGS2)	0.207923	1.65E-06
M2 Macrophage	CD163	0.116028	0.007965
	VSIG4	0.07562	0.084339
	MS4A4A	0.054135	0.216913
Neutrophils	CD66b(CEACAM8)	-0.20892	1.47E-06
	CD11b(ITGAM)	-0.08721	0.04642
	CCR7	-0.15346	0.000434
Natural Killer cell	KIR2DL1	-0.01247	0.776283
	KIR2DL3	-0.08083	0.064978
	KIR2DL4	-0.01864	0.67085
	KIR3DL1	-0.14488	0.000901
	KIR3DL2	-0.10606	0.015345
	KIR3DL3	-0.0838	0.055689
	KIR3DS4	-0.06063	0.166625
Dendritic cell	HLA-DPB1	-0.11142	0.010853
	HLA-DQB1	-0.06576	0.133485
	HLA-DRA	-0.02868	0.513213
	HLA-DPA1	-0.03195	0.466375
	BDCA-1(CD1C)	-0.08503	0.052187
	BDCA-4(NRP1)	0.377634	3.88E-19
	CD11c(ITGAX)	-0.01675	0.702671

Description	Gene markers	HNSC-MYO1B	
		correlation	Pvalue
Th1	T-bet(TBX21)	-0.15208	0.000489
	STAT4	0.141827	0.001158
	STAT1	0.337295	2.35E-15
	IFN-γ(IFNG)	-0.07691	0.07916
	TNF-α(TNF)	0.195449	6.85E-06
Th2	GATA3	0.03441	0.432727
	STAT6	0.137826	0.001597
	STAT5A	-0.10372	0.017766
	IL13	-0.05288	0.227744
Tfh	BCL6	-0.16356	0.000175
	IL21	-0.05701	0.193446
Th17	STAT3	0.104317	0.017118
	IL17A	-0.07912	0.070876
Treg	FOXP3	0.038963	0.374319
	CCR8	0.150401	0.000565
	STAT5B	0.140201	0.001321
	TGFβ(TGFB1)	0.513441	1.89E-36
T cell exhaustiion	PD-1(PDCD1)	-0.14815	0.000685
	CTLA4	-0.03779	0.388933
	LAG3	-0.05602	0.201297
	TIM-3	0.026495	0.545847
	GZMB(HAVCR2)	-0.06728	0.124717

Table S1: TIMER database query for correlation of MYO1B genes with 16 markers of immune cells infiltrating tumors.

Sequences of gene-specific primers used for real-time RT-qPCR

Gene	Forward primer (5'-3')	Reverse primer (5'-3')
TPRG1-AS1	TCAAAAGGCCTTGATCCCGA	AAGGACTCTGCTTCATGGGTG
MYO1B	GCTCTGGTGTGGAGGTCCTA	CGTTGCTTCCTCAGGTCTTC
miR-363-3p	AATTGCACGGTATCCATCTGTA	CTCAACTGGTGTCGTGGA
GAPDH	GTCTCCTCTGACTTCAACAGCG	ACCACCCTGTTGCTGTAGCCAA
U6	CTCGCTTCGGCAGCACA	AACGCTTCACGAATTTGCGT

Table S2: Sequences of gene-specific primers used for real-time RT-qPCR
